# Flight Speeds among Bird Species: Allometric and Phylogenetic Effects

**DOI:** 10.1371/journal.pbio.0050197

**Published:** 2007-07-17

**Authors:** Thomas Alerstam, Mikael Rosén, Johan Bäckman, Per G. P Ericson, Olof Hellgren

**Affiliations:** 1 Department of Animal Ecology, Lund University, Lund, Sweden; 2 Department of Vertebrate Zoology, Swedish Museum of Natural History, Stockholm, Sweden; University of Oxford, United Kingdom

## Abstract

Flight speed is expected to increase with mass and wing loading among flying animals and aircraft for fundamental aerodynamic reasons. Assuming geometrical and dynamical similarity, cruising flight speed is predicted to vary as (body mass)^1/6^ and (wing loading)^1/2^ among bird species. To test these scaling rules and the general importance of mass and wing loading for bird flight speeds, we used tracking radar to measure flapping flight speeds of individuals or flocks of migrating birds visually identified to species as well as their altitude and winds at the altitudes where the birds were flying. Equivalent airspeeds (airspeeds corrected to sea level air density, *U*_e_) of 138 species, ranging 0.01–10 kg in mass, were analysed in relation to biometry and phylogeny. Scaling exponents in relation to mass and wing loading were significantly smaller than predicted (about 0.12 and 0.32, respectively, with similar results for analyses based on species and independent phylogenetic contrasts). These low scaling exponents may be the result of evolutionary restrictions on bird flight-speed range, counteracting too slow flight speeds among species with low wing loading and too fast speeds among species with high wing loading. This compression of speed range is partly attained through geometric differences, with aspect ratio showing a positive relationship with body mass and wing loading, but additional factors are required to fully explain the small scaling exponent of *U*_e_ in relation to wing loading. Furthermore, mass and wing loading accounted for only a limited proportion of the variation in *U*_e_. Phylogeny was a powerful factor, in combination with wing loading, to account for the variation in *U*_e_. These results demonstrate that functional flight adaptations and constraints associated with different evolutionary lineages have an important influence on cruising flapping flight speed that goes beyond the general aerodynamic scaling effects of mass and wing loading.

## Author Summary

Analysing the variation in flight speed among bird species is important in understanding flight. We tested if the cruising speed of different migrating bird species in flapping flight scales with body mass and wing loading according to predictions from aerodynamic theory and to what extent phylogeny provides an additional explanation for variation in speed. Flight speeds were measured by tracking radar for bird species ranging in size from 0.01 kg (small passerines) to 10 kg (swans). Equivalent airspeeds of 138 species ranged between 8 and 23 m/s and did not scale as steeply in relation to mass and wing loading as predicted. This suggests that there are evolutionary restrictions to the range of flight speeds that birds obtain, which counteract too slow and too fast speeds among bird species with low and high wing loading, respectively. In addition to the effects of body size and wing morphology on flight speed, we also show that phylogeny accounted for an important part of the remaining speed variation between species. Differences in flight apparatus and behaviour among species of different evolutionary origin, and with different ecology and flight styles, are likely to influence cruising flight performance in important ways.

## Abstract 3

Measurement of flight speeds of 138 species of bird reveals that mass and wing loading do not scale according to aerodynamic theory but vary significantly depending on phylogeny.

## Introduction

According to fundamental aerodynamics the lift force (*L*) generated on a wing is related to flight speed (*U*) as:

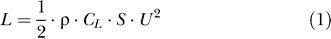
where ρ is air density, *S* is wing area, and *C*_L_ is the lift coefficient [1–3]. In horizontal cruising flight *L* balances the weight (*m × g*), and aircraft as well as animals are expected to fly at or near a value of *C*_L_ giving the maximum efficient lift-drag ratio. Provided that this value of *C*_L_ is about equal among bird species (as required for dynamical similarity) [1], it follows that cruising flight speed among bird species is expected to scale with body mass and wing loading (*Q* = *m × g*/*S*) as *U* ∝ *m* ^1/6^ and *U* ∝ *Q* ^1/2^, respectively (with the former proportionality based also on the assumption of geometrical similarity; i.e., *S* varies with *m* ^2/3^). These scaling rules have also been used to compare general speeds of a wide range of flyers, from the smallest insects to the largest aircraft [1,4–6].

In the absence of reliable measurements of the airspeed of different bird species in long-distance cruising (migration) flight, theoretically derived flight speeds for species of different mass and wing morphology have been used to explore these scaling rules [4,5,7–10]. Deviations from the expected scaling exponent in relation to mass have been found because of departures from geometrical similarity—larger birds often tend to have proportionately larger wing area and span [2,5,9–11]. There are additional possible reasons, besides departure from geometrical similarity, why bird flight speeds may deviate from the aerodynamic scaling rules. Flight adaptations related to the birds' ecology and phylogeny may have consequences for their cruising flight speeds, and different flight modes (continuous or intermittent flapping) may constrain the birds' speeds [2,10].

A full evaluation of the applicability of aerodynamic scaling rules must be based, not on theoretically derived speeds, but on empirical measurements of airspeeds of a wide variety of bird species in natural cruising flight. Here, we present tracking radar measurements of flight speeds of 138 species from six main monophyletic groups [12], which were analysed in relation to biometry *(m, S,* and wingspan *b)* and evolutionary origin (as reflected by phylogenetic group). All speeds reported here refer to flapping flight at cruising speeds of birds on migration. By restricting the analysis to migration flight we expect the birds to fly at an airspeed close to that associated with maximum lift-drag ratio [13]. All speeds designate equivalent airspeeds (*U*_e_) corrected to sea level air density [14,15].

## Results

**Figure 1:**
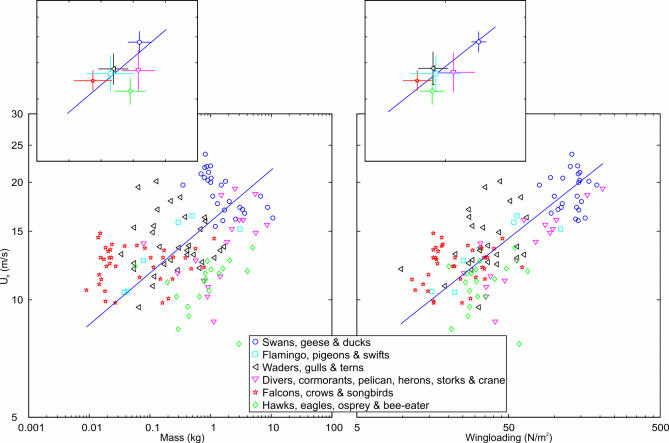
Bird Flight Speeds (*U*_e_; m/s) Plotted in Relation to Body Mass (kg) and Wing Loading (N/m^2^) for 138 Species of Six Main Monophyletic Groups The lines show the scaling relationships *U*_e_ = 15.9 × (mass)^0.13^ and *U*_e_ = 4.3 × (wing loading)^0.31^ as calculated by reduced major axis regression for all species (Table 1). All axes are in logarithmic scale. Inserts show means (± standard deviations) for the six main phylogenetic groups in relation to these scaling lines. Species of the same group tend to fly at similar speeds, and phylogenetic group is an important factor to account for the variation in *U*_e_.

Relationships between *U*_e_ and *m* and *Q* for all different species are plotted in Figure 1, with the lines showing the allometric relations according to reduced major axis regressions (Table 1). Mean airspeeds among the 138 species ranged between 8 and 23 m/s. Birds of prey, songbirds, swifts, gulls, terns, and herons had flight speeds in the lower part of this range, while pigeons, some of the waders, divers, swans, geese, and ducks were fast flyers in the range 15–20 m/s. Cormorants, cranes, and skuas were among the species flying at intermediary speeds, about 15 m/s. The diving ducks reached the fastest mean speeds in our sample, with several species exceeding 20 m/s, up to 23 m/s (Protocol S1).

**Table 1:**
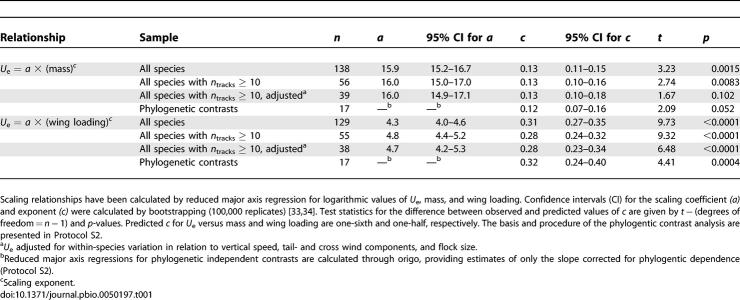
Allometric Relationships between Bird Flight Speed (*U*_e_; m/s) and Body Mass (kg) and between *U*_e_ and Wing Loading (N/m^2^)

The scaling analyses at the species level are robust against possible biases from few tracks per species and from within-species variation in speed (see Materials and Methods and Table 1). Because species do not represent an evolutionary independent data point, we also calculated scaling exponents by analysis of independent phylogenetic contrasts [16] according to the procedure and phylogeny [12] presented in Protocol S2. We used the well-resolved molecular phylogeny by Ericson et al. [12] for our phylogenetic analyses and classifications. The scaling results corrected for phylogenetic dependence agreed very closely with the exponents calculated on the species level (Table 1), demonstrating that the scaling exponents for *U*_e_ in relation to *m* as well as *Q* (0.12 and 0.32, respectively; phylogenetic contrast analysis) were smaller than the predicted values of 0.17 and 0.50, respectively. For the scaling of *U*_e_ versus *m*, the difference from the predicted value was at the significance level of 0.05 for the phylogenetic contrasts analysis, and the difference was not statistically significant for the sample of speeds adjusted for within-species variation (Table 1).

Within the different main phylogenetic groups (species level) as defined in Protocol S1 (see Figure 1), the scaling exponents of *U*_e_ in relation to *m* were significantly smaller than the predicted value of 0.17 among two of the groups. Swans/geese/ducks showed a remarkable negative scaling exponent of −0.15 (difference from prediction *t* = 13.40, degrees of freedom (df) = 25, and *p* < 0.0001), and falcons/crows/songbirds showed a scaling exponent of 0.08 that was clearly smaller than expected (*t* = 6.01, df = 37, and *p* < 0.0001). For the other four groups, the scaling exponents ranged between 0.12 and 0.20 and were not significantly different from the predicted value (*p* > 0.2). The corresponding scaling exponents of *U*_e_ in relation to *Q* differed significantly from the predicted value of 0.5 among three of the groups, flamingo/pigeons/swifts (exponent 0.28, *t* = 3.22, df = 5, and *p* = 0.023), divers/cormorants/pelican/herons/storks/crane (exponent 0.36, *t* = 2.59, df = 15, and *p* = 0.021), and falcons/crows/songbirds (exponent 0.28, *t* = 4.88, df = 37, and *p* < 0.0001). For the remaining three groups, the scaling exponents ranged between 0.42 and 0.54 and were not significantly different from the predicted value (*p* > 0.4).

To determine if there were geometrical differences in wing shape associated with differences in mass and wing loading, we investigated whether or not aspect ratio scaled significantly with *m* and *Q*. Aspect ratio is a dimensionless measure of wing shape (=*b* ^2^ */S*). We found significant departures from isometry with aspect ratio scaling positively to *m* as well as *Q* (*p* < 0.01 on the basis of all species [*n* = 129] and *p* < 0.05 on the basis of independent phylogenetic contrasts [*n* = 17], for both scaling relationships).

**Figure 2:**
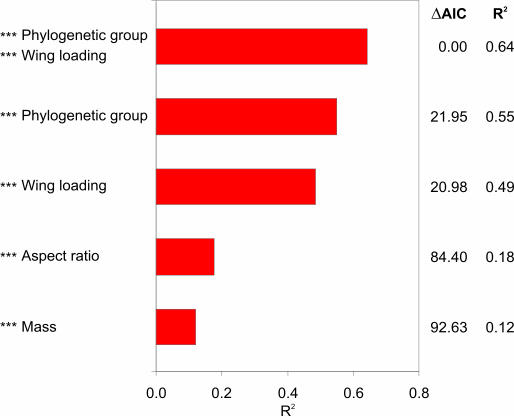
Explanation of the Variation in Mean Flight Speeds (*U*_e_; m/s) among Bird Species by Different Combinations of Variables and Factors The explanatory power (adjusted *R* ^2^) of different General Linear Models with significant independent variables (***, *p* < 0.001) is illustrated. Phylogenetic group and wing loading emerge as key factors to account for the variation in flight speed among bird species. General Linear Models for all different combinations of body mass, wing loading, aspect ratio, and phylogenetic group were calculated, except combinations including both body mass and wing loading (because of the interdependence between these variables). Complex models (including combinations of variables) are presented only if the AIC improved from models based on single independent variables [17]. This applied only to the model incorporating both phylogenetic group and wing loading. ΔAIC indicates the difference in AIC score from the most effective model (with ΔAIC = 0). Test statistics were as follows (in parentheses) for model including mass (*F*_1,136_ = 20.0, *p* < 0.001), aspect ratio (*F*_1,127_ = 28.6, *p* < 0.001), wing loading (*F*_1,127_ = 122.6, *p* < 0.001), phylogenetic group (*F*_5,132_ = 34.5), and phylogenetic group plus wing loading (*F*_6,122_ = 39.6, *p* < 0.001).

We also investigated the explanatory power of *m, Q,* aspect ratio, and phylogenetic group to account for the variation in *U*_e_ (Figure 2). Mass accounted for only a small fraction of the variation in flight speed while, as expected, speed was much more closely correlated with wing loading. There was a significant positive correlation between *U*_e_ and aspect ratio, but aspect ratio provided no improvement of general linear models (based on Akaike information criterion [AIC] [17]) when combined with *Q* or phylogenetic group.

A most potent factor to account for the variation in *U*_e_ was phylogenetic group; species of the same group tended to fly at similar characteristic speeds. The groups including birds of prey and herons had on average slow flight speeds for their mass and wing loading, while the average speed for groups including songbirds and shorebirds fell above the overall scaling lines (Figure 1). Main phylogenetic group alone accounted for a substantial proportion of the variation in *U*_e_ (adjusted *R* ^2^ = 0.55), and a general linear model including both *Q* and phylogenetic group was the most satisfactory model according to AIC (with adjusted *R* ^2^ = 0.64; Figure 2).

Our estimates of the explanation provided by the phylogenetic component, according to Figure 2, are likely to be conservative because of the broad grouping across the entire modern bird phylogeny. If tighter monophyletic groups at the family level were used (20 phylogenetic groups), phylogenetic group accounted for a fraction as high as 0.68 (adjusted *R* ^2^; *F*_19,118_ = 16.4, and *p* < 0.001) of the variation in *U*_e_, and for a model including both phylogenetic group and *Q* this fraction increased to 0.71 (adjusted *R* ^2^; *F*_20,108_ = 16.4, and *p* < 0.001). However, these models had positive ΔAIC-values (+8.1 and +28.8, respectively) in relation to the best model in Figure 2 and were thus less satisfactory when considering fit and complexity in combination [17].

## Discussion

Two main results emerged from our analyses; (1) that flight speeds among bird species scaled significantly differently with mass and wing loading than predicted from basic aerodynamic principles and (2) that phylogenetic group contributed in a highly significant way to explain the considerable variation in bird flight speeds that remained, even after the biometrical dimensions of the bird species had been taken into account.

### Scaling of Flight Speed

The scaling exponents fell below predicted values for both of the tested relationships, for *U*_e_ versus *m* as well as *U*_e_ versus *Q*. Predicted scaling exponents were based on the assumptions of geometrical and dynamical similarity. Could deviations from one or both of these assumptions explain our results? Earlier studies have demonstrated that bird species are not, on average, geometrically identical, but larger species tend to have proportionately longer wingspans and larger aspect ratios [2,5,10]. This was confirmed for the sample in the present study with aspect ratio scaling significantly positively to *m* as well as *Q*.

An overall scaling exponent of 0.14 for flight speed versus body mass was calculated for theoretical flight speeds after taking the slight positive allometry in wing size into account for a large sample of bird species [9]. This fits well with the corresponding exponent for observed speeds in this study, making departure from geometrical similarity a likely explanation for this result. The negative scaling exponent of *U*_e_ in relation to *m* for the swans, geese, and ducks may be an effect of a reduced flight power margin with increasing size restricting the largest flyers like swans to fly close to the minimum power speed rather than at the faster speed associated with maximum effective lift-drag ratio [18,19]. Such constrained flight speeds for the largest flyers will also have the effect of reducing the overall scaling exponents, thus providing another contributory explanation for the observed results in this study.

Dynamical similarity is reflected by Reynolds number, which will differ between bird species in proportion to their size (length dimension) and speed [20]. Reynolds number shows a 15-fold range among the species in our sample (ranging from approximately 25,000 to 375,000 based on mean wing chord, *S*/*b,* as length measurement). Such a range of Reynolds number may well be large enough to give rise to significant departures from dynamical similarity. The main expected consequence would be a reduced coefficient of frictional drag for birds with large Reynolds number (i.e., large and fast birds) leading to an increased optimal cruising speed among these species [14,20]. Thus, such a departure from dynamical similarity is expected to show up as an augmented scaling exponent for *U*_e_ versus *m* (and also for *U*_e_ versus *Q*), rather than a scaling exponent lower than expected as in this analysis.

In view of the opposite effects on scaling exponents of departures from geometrical and dynamical similarity, respectively [1], we conclude that only the departure from geometric similarity can explain why the scaling exponent for *U*_e_ versus *m* falls significantly below one-sixth among birds in cruising migratory flight.

Do geometrical differences provide a sufficient explanation also for the fact that the scaling exponent for *U*_e_ versus *Q* fell clearly below the expected value of one-half? One way to evaluate this is to calculate the scaling exponent for flight speed versus span loading (*m* × *g/b* ^2^, where *b* is wingspan). Span loading is equivalent to wing loading divided by the aspect ratio, and for birds differing in their geometric wing shapes cruising flight speed is expected to scale most closely with the square root of span loading (under geometrical similarity flight speed is predicted to scale with the same exponent of one-half versus both span loading and wing loading) [5].

The scaling exponent for *U*_e_ versus span loading (species level, exponent 0.36 with 95% confidence interval 0.31–0.40, *n* = 129 and phylogenetic contrasts, exponent 0.37 with 95% confidence interval 0.26–0.48, *n* = 17) exceeded that versus *Q* (with corresponding exponents of 0.31 and 0.32, respectively, Table 1) although still falling significantly below the predicted value of one-half. This suggests that the geometrical differences explain part, but not all, of the discrepancy between observed and expected scaling of *U*_e_ versus *Q*. Departure from dynamical similarity will, in its most simple form (as reflected by differences in Reynolds number), contribute to an augmented rather than reduced scaling exponent in relation to that predicted and can therefore not provide any useful additional explanation in this case (see above). Still, dynamical differences of other kinds may exist for reasons that are notoriously difficult to predict for flapping flight. Future studies of vortex patterns associated with flapping flight of different species will be important to demonstrate possible dynamical differences between species (see below).

We suggest that the unexpectedly small scaling exponent for *U*_e_ versus *Q* may be the result of general evolutionary forces acting to increase cruising speeds for species with the lowest wing loadings and reduce speeds for species with the highest wing loadings. The bird species in our analysis show approximately a 10-fold difference in their range of *Q* (from about 15 to 150 N/m^2^, Figure 1). With an observed scaling exponent for flight speed of 0.31, this range of *Q* is associated with a 2-fold (10^0.31^ = 2.0) difference in flight speed. However, with a predicted scaling exponent of 0.5 we would have expected more than a 3-fold difference in cruising speed (10^0.5^ = 3.2). Given that birds with low *Q* (about 15 N/m^2^) fly at a speed about 10 m/s (as observed), species with high *Q* (about 150 N/m^2^) would fly at 32 m/s according to the general aerodynamic scaling rules. This may well be impracticably fast and difficult to reconcile with flight performance in situations of start, landing, flock manoeuvres, etc. Conversely, given that birds with high *Q* fly at a speed about 20 m/s (as observed), species with low *Q* would fly at only about 6 m/s according to the general aerodynamic scaling rules. Such very slow speeds will be disadvantageous because of sensitivity to wind, vulnerability to predation, etc. Hence, it seems reasonable to expect that there are evolutionary forces operating to compress the range of cruising flight speeds among bird species [5] and thus reducing the scaling exponent for *U*_e_ versus *Q*. This compression of the range of flight speeds is attained partly through general geometrical differences between species (larger aspects ratios among species with larger mass and wing loading, as discussed above), but additional unknown mechanisms, perhaps associated with different kinematics of flight or different muscle operation between species, seem to be required to fully explain the restricted range of flight speeds among bird species.

Bounding flight seems to be a mode for small birds (mainly passerines) to mitigate the costs of fast flight [1,2,10,21], while flap-gliding, used by many raptors, is associated with a reduction in cruising flight speed [21]. Both of these styles of intermittent flight are used by species with low or intermediate *Q* (Figure 1), and, having opposite effects on flight speed, they are unlikely to provide a sufficient explanation for the low scaling exponent of *U*_e_ versus *Q* among bird species as a whole.

### Variability of Flight Speeds

Dimensional analyses have demonstrated that scaling relationships between wing loading and total mass differ significantly between different types of birds [5,10]. The expected consequence of this is that wing loading will be a more reliable predictor of flight speed, explaining more of the variation in flight speeds among bird species than body mass [1,5]. This expectation was fully confirmed in the present study, with *Q* accounting for almost half of the variation in *U*_e_ between species, while *m* explained only 12% of this variation (Figure 2). However, our findings that *Q* still left a large part of the variation in flight speed unexplained and that phylogenetic group accounted for a significant fraction of this remaining variation were unexpected from earlier analyses based on theoretically calculated flight speeds [5,10].

What are the causes for the discrepancies in flight speed between phylogenetic groups? Differences in flight mode and the use of bounding flight by many passerines have been suggested as explanations for important group-specific deviations from aerodynamic predictions of optimal bird flight speeds [15]. We provisionally assigned, based on our own field experience, the different bird species to three main modes of flapping flight; (1) continuous flapping (e.g., shorebirds and ducks), (2) intermittent flapping with short gliding phases (raptors, swifts, and swallows), and (3) bounding flight (many but not all passerines use this mode of intermittent flapping with phases of wing folding). *U*_e_ differed significantly between flyers in these three categories (*p* < 0.001, adjusted *R* ^2^ = 0.26, and *F*_2,135_ = 25.1), and the explanatory power of a model incorporating both flight mode and *Q* was high (*p* < 0.001, adjusted *R* ^2^ = 0.60, and *F*_3,125_ = 64.5). This suggests that difference in flight mode is one element affecting the characteristic cruising flight speeds among phylogenetic groups.

Depending on their ecological life style and foraging, birds are adapted to different aspects of flight performance, e.g., speed, agility, lift generation, escape, take-off, cost of transport, and power [2,10]. These adaptations are likely to have implications for the flight apparatus (anatomy, physiology, and muscle operation) and the flight behaviour that may constrain the cruising flight speed. The variations in power-versus-speed relationships between different species [22] and in muscle efficiency (conversion from metabolic power input to mechanical power output) with mass and flight speed [23,24] may be related to such differential complex flight adaptations among birds. Constraints on flight speed may also be associated with differences in fluid dynamics and vortex patterns, hereto investigated only for a few species [25–27]. Variable airspeeds may still be associated with high power efficiency if accompanied with the proper variation in wing stroke frequency and amplitude [28,29].

Species flying at comparatively slow cruising speeds frequently use thermal soaring (raptors and storks), are adapted for hunting and load carrying (raptors), or for take-off and landing in dense vegetation (herons). Associated with these flight habits they have a lower ratio of elevator (supracoracoideus) to depressor (pectoralis) flight muscle (particularly low among birds of prey) compared with shorebirds and anatids [2]. We suggest that functional differences in flight apparatus and musculature among birds of different life and flight styles (differences often associated with evolutionary origin) have a significant influence on the birds' performance and speed in sustained cruising flight. Thus, our results strongly indicate that there is a diversity of cruising flight characteristics among different types of birds over and above the general scaling effects of mass and wing loading that remains to be investigated and understood, aerodynamically [30], kinematically [26,31], physiologically [22], as well as ecologically [2,10].

## Materials and Methods

### Tracking radar measurements.

Our main dataset, based on tracking radar measurements in Sweden and the Arctic 1979–1999, consists of 1,399 tracks of 102 identified species, with a mean track time of 369 s (range 20–2,220 s). Altitudes ranged from sea level to 3,600 m. Number of tracks for each species ranged between one and 240, and mean *U*_e_ (with SD), vertical speed as well as information about number of tracks, track time, and biometry data are given for each species in Protocol S1.

An extensive additional dataset of equivalent airspeeds of identified birds, obtained by similar tracking radar techniques, has been published from the work of Bruno Bruderer and his research group in Switzerland, Germany, Israel, and Spain [15]. Flight speed data from tracks of birds in natural migratory flight (excluding released birds and soaring flight) were incorporated into our analysis. This additional dataset comprised 64 species, and with 28 species shared between the two sets of data, the combined data added up to a total of 138 species (Protocol S1). Mean *U*_e_ for the shared species were not significantly different between the two sets (paired sample t-test, *t* = 1.28, and *p* = 0.21), and we used weighted (according to the number of tracks) overall mean *U*_e_ for these species in our analyses.

The bulk of flight speed data were measured 1979–1999 by tracking radar studies at five sites in southern Sweden and on two expeditions by icebreaker to the Arctic (for detailed methods see [19,32]). Targets were identified to species and flock sizes through telescopes simultaneously with radar registrations providing computer readings of range, elevation, and bearing to the target usually every 10 s with the radar in automatic tracking mode. All flight speeds have been corrected for the influence of wind by subtraction of the wind vector at the altitude where the birds were flying from the ground speed vector of the birds. Winds were measured by releasing and tracking hydrogen/helium-filled balloons carrying a radar reflector. Mean airspeed, altitude, and vertical flight speed were calculated for each track, excluding segments with a convoluted flight path. Altitudes were corrected in relation to sea level by adding the altitude of the radar antenna (10–185 m above sea level at the different sites), and true airspeeds were reduced to equivalent airspeeds (*U*_e_) referring to sea level air density, according to the standard atmosphere change in air density with altitude [14,15].

### Scaling calculations and statistical analyses.

Reduced major axis regressions [16] for the scaling relationships between *U*_e_ and *m* and *Q,* respectively, were performed in Matlab, with calculations of confidence intervals by bootstrapping [33]. Calculations of reduced major axis regressions based on phylogenetic independent contrasts are further described in Protocol S2. We checked for possible bias arising as a consequence of including species with only one or a few tracks, by restricting the calculations to species with at least five or ten tracks. The results remained the same, as exemplified for the sample of 56 species with ≥10 tracks in Table 1. For 39 of the species with ≥10 tracks, we could account for the within-species variation of *U*_e_ in relation to vertical flight speed, head- and side-wind components, and flock size by multivariate regression (statistically significant influences were found in 26 of these 39 species; unpublished data). Restricting the analysis to intercept values of *U*_e_ for these 39 species (corrected to zero vertical speed, zero wind, and a flock size of one from the multiple regression equations of significant variables for each species) still gave the same scaling result (Table 1). General Linear Models (Figure 2) [34] were calculated with *U*_e_ as dependent variable. Logarithmic values were used for *U*_e_, *m,* and *Q*. Phylogenetic group and flight mode (limited analysis of this provisionally estimated variable) were treated as fixed factors. Complex models (different combinations or interactions of mass, aspect ratio, and phylogenetic group or of wing loading, aspect ratio, and phylogenetic group) were presented in Figure 2 only if AIC improved from that of models with single independent variables [19].

## Supporting Information

Protocol S1 Supplementary List of Flight Speeds and Biometry of Bird Species (173 KB PDF)

Protocol S2 Supplementary Information on Phylogenetic Tree, Taxon Sampling, and Analysis of Independent Phylogenetic Contrasts (29 KB PDF)

## Abbreviations

AIC: Akaike information criterion
df: degrees of freedom
