# Supplementary material for: Flight Speeds among Bird Species: Allometric and Phylogenetic Effects
Source: PLoS Biol. 2007 Jul 17;5(8):e197. doi: 10.1371/journal.pbio.0050197 (PMC1914071; doi:10.1371/journal.pbio.0050197)
Supplement: Supplementary file 1 [file pbio.0050197.sd001.pdf]

Protocol S1
Supplementary list of flight speeds and biometry of bird species

The table gives flight speeds and biometry for 138 species of birds in continuous flapping flight tracked by radar. Tracking details are only given for the novel data collected by the T.A. group at Lund University (102 species). Data from Bruderer and Boldt (2001, 64 species, indicated by •) are presented for their subset of birds in continuous flapping flight.

The following data referring to speeds are listed: mean equivalent airspeed (U_e), standard deviation of U_e, mean vertical speed (U_z), number of tracks (n) and total track time for the radar recordings. For the data from Bruderer and Boldt (2001) only mean U_e and number of tracks, included into the present analysis, are given (for further details see Bruderer & Boldt 2001).

Data on biometry is based on a review of 33610 measurements of body mass, wingspan or wing area, where all measurements of a given parameter refer to unique birds. The contribution of each source on the species level was: Dunning (1993, 135 species), Magnan (1922, 87), Bruderer and Boldt (2001, 82), Pennycuick (1999, 57), Greenewalt (1962, 55), Hedenström, A. (unpubl., 31), Hedenström (1994, 14), Strandberg, R. (unpubl., 6), Cramp et al. (1977-94, 2), Hedenström (1999, 1), Hedenström (1992, 1) and Rosén, M. (unpubl., 1). Weighted averages are given in the table and the total mean is the mean of all species.

The following information referring to biometry are listed: Number of body mass measurements, mean total body mass of a fresh killed or live bird (kg), number of wing span measurements, mean wing span (b; maximum wingtip to wingtip distance on a bird in the hand, in metres), number of wing area measurements, wing area (S; projected area of both wings when fully stretched including the area of the body between the wings, m^2). Data was selected to compare with the methods of measuring birds by Pennycuick (1999) and was used as is from all authors except from Greenewalt (1962: Table 8). Here, the wing area was corrected (increased) by using ‘wing spread’ instead of ‘length of both wings’ given a constant aspect ratio (b^2/S), and only ‘wing spread’ was used as a measure of wingspan (cf.

Greenewalt, 1962). Species are aranged into six main phylogenetic groups according to the phylogeny of Ericson et al. (2006).

References in this protocol:

Bruderer, B. & Boldt, A. (2001) Flight characteristics of birds: 1. radar measurements of speeds. *Ibis* **143**:178-204.

Cramp, S., Simmons, K.E.L & Perrins, C.M. , eds. (1977-94) *The Birds of the Western Palearctic*, vol 1-9 (Oxford University Press).

Ericson, P.G.P., Anderson, C.L., Britton, T., Elzanowski, A., Johansson, U.S., Källersjö, M., Ohlson, J.I., Parsons, T.J., Zuccon, D. & Mayr, G. (2006) Diversification of Neoaves: integration of molecular sequence data and fossils. *Biol. Lett.* **2**: 543-547.

Dunning, J.B. (1993) *CRC Handbook of Avian Bodymasses* (CRC Press, Boca Raton).

Greenewalt, C.H. (1962) Dimensional relationships for flying animals. *Smithsonian miscellaneous collections* **144**(2): 1-46.

Hedenström, A. & Alerstam, T. (1992) Climbing performance of migrating birds as a basis for estimating limits for fuel-carrying capacity and muscle work. *J. exp. Biol.* **164**: 19-38.

Hedenström, A. (1994) Flight strategies in Arctic Birds. In *Tundra Ecology 1994: A Cruise Report* (Swedish Polar Research Secretariat, Stockholm).

Hedenström, A., Rosén, M., Åkesson, S. & Spina, F. (1999) Flight performance during hunting excursions in the Eleonora's falcon *Falco eleonorae*. *J. exp. Biol.* **202**: 2029-2039.

Magnan, A. (1922) Les caractéristiques des oiseaux suivante le mode de vol. *Ann. Sci. Nat.* **10**(5): 125-134.

Pennycuick, C.J. (1999) *Measuring birds' wings for flight performance calculations*, 2nd edition (Boundary Layer Publications, Bristol).

Taxon	Species	U_e (m/s)	sd (m/s)	U_z (m/s)	N (tracks)	Track-time (s)	N (mass)	Mass (kg)	N (b)	b (m)	N (S)	S (m^2)
Swans, geese & ducks	*Cygnus olor*	16.2	1.6	0.1	24	8540	115	10.597	21	2.30	9	0.6504
	Cygnus columbianus	18.5		0.4	1	440	167	6.637	12	1.98	11	0.4608
	Cygnus cygnus	17.3	2.0	-0.1	10	3969	25	8.689	19	2.29	18	0.6045
	Anser fabalis	17.3	2.7	0.0	44	14600	127	3.035	1	1.62	1	0.2675
	Anser albifrons	16.1	2.0	0.1	5	1255	169	2.582	1	1.41	1	0.1835
	Anser anser	17.1	2.6	0.0	21	10100	176	3.326	7	1.55	6	0.3079
	Branta canadensis	16.7		0.2	1	180	395	3.628	4	1.69	4	0.3717
	Branta leucopsis	17.0	2.2	0.0	22	10740	620	1.705	1	1.08	1	0.1150
	Branta bernicla	17.7	2.8	0.1	97	45450	792	1.306	3	1.01	3	0.1129
	Tadorna tadorna	15.4		0.0	1	120	16	1.193				
	Anas penelope	20.6	2.1	0.1	36	15300	77	0.783	11	0.82	11	0.0814
	Anas crecca	19.7	4.0	0.2	9	3050	281	0.348	6	0.59	5	0.0428
	Anas platyrhynchos	18.5	2.3	0.3	22	7470	5871	1.082	24	0.88	24	0.1062
	Anas acuta	20.6	2.6	0.4	6	1920	294	1.024	2	0.90	2	0.0879
	Aythya ferina	23.6		0.7	1	190	322	0.823	1	0.77	1	0.0615
	Aythya fuligula	21.1	1.1	0.7	3	670	1044	0.694	1	0.71	1	0.0474
	Aythya marila	21.3		1.1	1	150	27	0.931	1	0.82	1	0.0621
	Somateria mollissima	17.9	2.4	0.0	240	79218	56	2.015	2	0.98	2	0.1310
	Somateria spectabilis	16.0	0.3	-0.2	2	740	183	1.591	1	0.93	1	0.1080
	Polysticta stelleri	21.9		-0.1	1	310	90	0.805				
	Clangula hyemalis	22.0	1.4	0.1	3	510	1299	0.874	2	0.71	2	0.0669
	Melanitta nigra	22.1	4.0	-0.1	14	5220	13	0.990	1	0.85	1	0.0679
	Melanitta fusca	20.1	4.7	0.0	32	10760	13	1.743	1	0.97	1	0.1010
	Bucephala clangula	20.3	3.8	0.3	10	4560	113	0.901	2	0.70	2	0.0598
	Mergus serrator	20.0	2.9	0.2	33	13430	37	1.004	2	0.87	2	0.0678
	Mergus merganser	19.7	1.1	0.1	2	420	26	1.489	2	0.93	2	0.0767
Flamingo, pigeons & swifts	*Phoenicopterus ruber* •	15.2			3		26	3.053	1	1.53	1	0.2715
	Columba oenas	15.8	2.0	0.1	8	1830	20	0.295	1	0.75	1	0.0532
	Columba palumbus	16.3	2.3	0.3	87	45920	522	0.490	3	0.75	3	0.0842
	Columba palumbus •	17.6			11							
	Apus apus	9.7	2.0	0.4	34	18570	230	0.038	14	0.40	13	0.0168
	Apus apus •	10.6			163							
	Apus pallidus •	10.5			33		661	0.042	1	0.44	1	0.0263
	Apus melba •	12.6			36		13	0.078	1	0.57	1	0.0304
Waders, gulls & terns	*Haematopus ostralegus*	13.0	2.5	0.2	19	9930	293	0.523	6	0.82	4	0.0810
	Charadrius hiaticula	19.5		0.4	1	130	84	0.064	9	0.41	9	0.0179
	Pluvialis dominica	13.7		0.2	1	790	60	0.145				
	Pluvialis squatarola	17.9	3.9	0.1	14	4870	37	0.219	6	0.62	6	0.0420
	Vanellus vanellus	12.8	1.3	0.0	14	3380	79	0.219	4	0.75	2	0.0744
	Vanellus vanellus •	11.9			2							
	Calidris canutus	20.1	1.9	-0.2	7	2990	42	0.128	20	0.50	19	0.0286
	Calidris alpina	15.3	1.9	0.5	36	13578	652	0.054	23	0.36	23	0.0156
	Philomachus pugnax	17.4	1.0	-0.1	3	1120	270	0.114	9	0.55	9	0.0388
	Philomachus pugnax •	13.6			3							
	Gallinago gallinago	17.1	2.7	0.0	22	5660	37	0.132	7	0.52	6	0.0476
	Limosa lapponica	18.3	2.1	0.2	15	7710	94	0.318	1	0.73	1	0.0520
	Numenius phaeopus	16.3	0.9	0.2	4	1260	66	0.383	1	1.07	1	0.1365
	Numenius arquata	16.3	2.3	0.3	16	7040	231	0.794	6	0.97	2	0.1182
	Tringa nebularia	12.3	3.3	0.1	5	1290	52	0.174	1	0.61	1	0.0406
	Tringa glareola	9.6	1.7	1.1	3	980	31	0.066	4	0.40	4	0.0203
	Arenaria interpres	14.9	0.7	0.7	2	630	35	0.111	11	0.47	11	0.0252
	Phalaropus lobatus	13.1		0.2	1	230	61	0.033	4	0.34	4	0.0146
	Phalaropus fulicarius	12.4	2.3	-0.1	6	1000	216	0.054	6	0.42	6	0.0196
	Stercorarius pomarinus	15.2	2.3	0.2	16	5320	128	0.688	3	1.18		
	Stercorarius parasiticus	13.8	2.2	-0.2	7	1500	45	0.438	17	1.06	16	0.1175
	Stercorarius longicaudus	13.6	1.9	0.6	3	1550	48	0.297	4	1.00	4	0.0891
	Larus minutus	11.5	0.1	0.2	2	610	87	0.118				
	Larus ridibundus	11.9	1.6	0.0	19	6190	352	0.283	28	0.97	28	0.0976
	Larus canus	13.4	2.9	0.1	36	14050	176	0.411	5	1.11	3	0.1246
	Larus fuscus	13.1	1.9	-0.2	11	3150	130	0.719	14	1.34	14	0.1934
	Larus fuscus •	11.9			11							
	Larus argentatus	12.8	1.8	0.0	18	7210	374	1.142	11	1.34	11	0.1968
	Larus glaucoides	15.9		-0.2	1	140	3	0.819				
	Larus hyperboreus	13.4	0.8	0.1	3	530	65	1.445				
	Larus marinus	13.7	1.2	-0.2	4	700	219	1.669	10	1.67	10	0.2881
	Rissa tridactyla	13.1	0.4	-0.1	2	660	411	0.408	6	0.96	6	0.0953
	Sterna caspia	12.1		0.6	1	210	84	0.655				
	Sterna paradisaea	10.9	0.9	0.4	2	500	262	0.110	1	0.80	1	0.0571
	Chlidonias leucopterus •	12.0			1		111	0.054	1	0.65	1	0.0545
Divers, cormorants, pelican, herons, storks & crane	*Gavia stellata*	18.6	3.9	0.0	7	3660	13	1.505	1	1.04	1	0.0890
	Gavia arctica	19.3	2.1	0.1	13	3710	5	2.543	1	1.20	1	0.1196
	Gavia adamsii	18.7	0.5	0.4	2	220	7	5.500				
	Phalacrocorax carbo	15.2	3.0	0.1	35	13410	62	2.227	9	1.40	9	0.2240
	Pelecanus onocrotalus •	15.6			1		7	8.504	5	2.91	4	0.9554
	Botaurus stellaris	8.8		1.0	1	340	4	1.133	2	1.26	2	0.2727
	Nycticorax nycticorax •	11.2			4		8	0.763	3	1.06	2	0.1589
	Ardeola ralloides •	11.7			1		24	0.287	1	0.86	1	0.1114
	Egretta alba •	10.2			2		27	0.888	6	1.44	4	0.2443
	Ardea cinerea	12.5	2.2	0.1	14	4840	34	1.439	3	1.73	3	0.3716
	Ardea cinerea •	11.2			49							
	Ardea purpurea •	10.8			12		3	0.906	1	1.35	1	0.2488
	Ciconia nigra •	16.0			6		2	3.000	1	1.50	1	0.2842
	Ciconia ciconia •	16.0			6		72	3.432	4	1.91	3	0.5326
	Plegadis falcinellus •	12.6			5		4	0.566	2	0.89	1	0.0986
	Platalea leucorodia •	14.1			7		9	1.857	2	1.30	2	0.2415
	Porzana porzana	13.9		-0.1	1	100	18	0.078	2	0.38	2	0.0236
	Grus grus	15.0	2.7	-0.2	12	6010	19	5.614	2	2.22	2	0.5855
	Grus grus •	13.6			1							

Falcons, crows & songbirds	*Falco naumanni* •	11.3			12		60	0.151	1	0.65	1	0.0611
	Falco tinnunculus	10.1		0.2	1	120	105	0.203	7	0.73	5	0.0708
	Falco vespertinus •	12.8			4		11	0.165	1	0.72	1	0.0728
	Falco subbuteo •	11.3			20		14	0.238	6	0.74	6	0.0667
	Falco eleonorae •	12.8			2		22	0.387	2	0.95	2	0.1041
	Falco peregrinus •	12.1			13		51	0.789	20	1.02	19	0.1257
	Lullula arborea •	9.8			1		962	0.027	1	0.29	1	0.0164
	Alauda arvensis	15.1	2.1	-0.4	15	2880	393	0.039	5	0.35	5	0.0207
	Alauda arvensis •	12.7			20							
	Riparia riparia	14.3		0.6	1	140	250	0.015	4	0.27	4	0.0096
	Riparia riparia •	11.3			9							
	Hirundo rupestris •	9.9			1		10	0.019	3	0.32	2	0.0116
	Hirundo rustica	10.0	2.0	-0.4	2	700	2343	0.016	6	0.32	6	0.0136
	Hirundo rustica •	11.3			108							
	Delichon urbica	9.7	0.5	-0.1	2	390	256	0.015	3	0.29	3	0.0104
	Delichon urbica •	11.0			76							
	Anthus trivialis	12.7	2.2	0.2	4	1170	438	0.022	2	0.27	2	0.0126
	Anthus trivialis •	12.0			4							
	Anthus pratensis •	10.5			6		232	0.018	4	0.26	4	0.0123
	Motacilla flava •	12.7			7		164	0.018	2	0.26	1	0.0103
	Motacilla alba	14.1	1.9	-0.3	5	610	101	0.021	8	0.26	8	0.0119
	Motacilla alba •	13.0			13							
	Prunella modularis •	12.2			1		541	0.020	14	0.21	14	0.0090
	Oenanthe oenanthe •	12.8			1		73	0.023	6	0.28	6	0.0143
	Turdus pilaris	13.0	2.9	0.2	15	6970	76	0.105	7	0.42	7	0.0299
	Turdus pilaris •	12.4			6							
	Turdus philomelos	11.0	2.1	0.4	6	2490	290	0.068	21	0.36	21	0.0218
	Turdus philomelos •	11.7			8							
	Turdus iliacus	13.8	1.8	0.3	6	1450	317	0.061	35	0.36	35	0.0223
	Turdus viscivorus	11.9	3.3	-0.4	3	600	21	0.114	2	0.44	2	0.0333
	Turdus viscivorus •	12.4			2							
	Parus ater	10.6	2.3	0.1	2	160	51	0.009	1	0.18	1	0.0073
	Parus major •	13.6			1		63	0.019	13	0.23	13	0.0109
	Garrulus glandarius	6.7	1.0	0.2	2	310	60	0.162	5	0.54	5	0.0644
	Garrulus glandarius •	12.9			2							
	Nucifraga caryocatactes	13.4	2.8	-0.1	2	370	72	0.173	2	0.58	2	0.0584
	Corvus monedula	12.5	2.6	0.0	13	3260	202	0.245	5	0.65	3	0.0684
	Corvus monedula •	14.7			3							
	Corvus frugilegus	11.5	1.2	0.1	16	4870	79	0.488	3	0.93	2	0.1380
	Corvus frugilegus •	13.0			8							
	Corvus corone	13.5	2.6	0.0	5	1260	147	0.566	6	0.91	5	0.1378
	Corvus corax	14.3	0.9	-0.1	5	1325	10	1.149	2	1.21	1	0.2472
	Sturnus vulgaris	16.2	4.3	-0.5	4	1360	2875	0.083	17	0.38	14	0.0244
	Sturnus vulgaris •	12.4			9							
	Fringilla coelebs	12.8	1.9	0.2	23	8410	169	0.022	18	0.26	18	0.0132
	Fringilla coelebs •	12.8			52							
	Fringilla montifringilla	15.0	2.9	-0.9	4	1110	63	0.024	13	0.27	13	0.0126
	Fringilla montifringilla •	11.6			2							
	Carduelis chloris •	12.2			1		25	0.028	6	0.25	6	0.0123
	Carduelis carduelis •	12.8			3		53	0.016	3	0.24	3	0.0093
	Carduelis spinus	14.5	1.6	-0.3	60	20150	59	0.014	9	0.21	9	0.0076
	Carduelis spinus •	12.4			2							
	Carduelis cannabina	14.8	4.5	-0.2	2	800	53	0.015	2	0.24	2	0.0095
	Pyrrhula pyrrhula	13.4		0.9	1	300	39	0.022	5	0.27	5	0.0136
Hawks, eagles, osprey & bee-eater	*Pernis apivorus*	12.5	2.3	-0.3	13	1860	28	0.778	10	1.26	10	0.2471
	Pernis apivorus •	10.1			16							
	Milvus migrans •	11.7			7		33	0.815	2	1.52	1	0.2805
	Milvus milvus	12.0	0.3	-0.5	2	400	17	1.012	3	1.66	3	0.3248
	Haliaeetus albicilla	13.6	0.9	-0.2	2	200	34	4.967	4	2.18	4	0.8824
	Neophron percnopterus •	12.6			3		11	2.062	2	1.65	1	0.3555
	Circus aeruginosus	11.2	1.6	-0.5	2	200	56	0.653	11	1.16	11	0.2040
	Circus aeruginosus •	10.1			22							
	Circus cyaneus	9.1	0.7	-0.2	2	180	363	0.433	3	1.10	3	0.1568
	Circus macrourus •	9.6			3		23	0.420	2	1.09	2	0.1483
	Circus pygargus •	8.4			1		22	0.291	3	1.09	3	0.1350
	Accipiter nisus	11.3	1.9	-0.1	12	3040	361	0.277	46	0.67	43	0.0768
	Accipiter nisus •	10.0			64							
	Accipiter brevipes •	11.1			57		10	0.195	1	0.70	1	0.0739
	Buteo buteo	11.6	2.7	-0.1	10	4200	491	0.885	15	1.24	14	0.2689
	Buteo buteo •	13.3			13							
	Buteo lagopus	10.5	0.0	-0.2	2	1120	279	0.943	8	1.35	7	0.3324
	Aquila pomarina •	11.7			2		38	1.391	1	1.47	1	0.5153
	Aquila nipalensis •	7.7			1		1	2.900	1	2.03	1	0.4853
	Aquila chrysaetos	11.9		-0.5	1	40	61	4.069	13	2.03	11	0.5970
	Hieraaetus pennatus •	11.3			2		24	0.828	1	1.11	1	0.2004
	Pandion haliaetus	13.3	2.7	0.0	5	1030	49	1.578	23	1.60	22	0.3201
	Pandion haliaetus •	11.4			10							
	Merops apiaster •	12.2			28		384	0.057	1	0.47	1	0.0273
	Mean (species)	14.1			17		232	1.065	6	0.91	6	0.1442
