# Supplementary material for: Flight Speeds among Bird Species: Allometric and Phylogenetic Effects
Source: PLoS Biol. 2007 Jul 17;5(8):e197. doi: 10.1371/journal.pbio.0050197 (PMC1914071; doi:10.1371/journal.pbio.0050197)
Supplement: Supplementary file 2 [file pbio.0050197.sd002.pdf]

Protocol S2
Supplementary information on phylogenetic tree, taxon sampling and analysis of independent phylogenetic contrasts

We calculated phylogenetic independent contrasts to explore cross-taxon relationships between U_e, mass and wing loading. The calculations require a phylogenetic hypothesis for the taxa involved, as well as estimates of the branch lengths in the tree. Our flight speed data (Appendix 1) have been obtained from a taxonomically wide range of species for which no comprehensive phylogeny exists.

To obtain a reasonable phylogenetic hypothesis to be used in the calculation of independent contrasts we selected 18 species for which taxonomic counterparts were studied by Ericson et al. (2006; the dataset is available at http://www.nrm.se/researchandcollections/zoology/vertebratezoology). As the taxonomic composition of that study differs somewhat from ours at the species level, we in some cases included in the independent contrasts analysis a species from our data set that is as taxonomically close as possible to that used by Ericson et al. (2006). In twelve cases we could use a conspecific or congeneric representative, and in four cases we could use another species of the same family (see Fig. S1 below). As representatives of the orders Anseriformes and Passeriformes, which both are undoubtedly monophyletic (see Ericson 1997, Livezey 1997, Barker et al. 2002, 2004, Ericson et al. 2002), we selected two species (*Branta leucopsis* and *Turdus pilaris*, respectively) that we considered as "typical" representatives of these orders in terms of size, proportions and flight speed.

The fact that we could not use exactly the same species as those in Ericson et al. (2006) in the contrast analysis is unlikely to affect the result. The phylogeny of Ericson et al. (2006) is very robust and the inclusion or exclusion of taxa in their analyses did not change the recovered phylogeny (Per Ericson pers. obs.). Also the branch lengths in the tree are unlikely to change dramatically by the use of alternative representatives at the family level since

Ericson et al. (2006) used slowly evolving nuclear markers to maximize resolution of ancient divergences. In the contrast analysis we thus assume that the number of substitutions, i.e. branch lengths, from the root to the tip in the tree, is equal for all members of a certain family. The branch lengths were estimated in a maximum-likelihood analysis using a general time-reversible model of evolution (Lanave et al. 1984, Rodriguez et al. 1990), taking into account the proportion of invariables sites, and a gamma distribution of the rates of substitution for the variable positions (GTR+I+G). The tree in Figure 1 in Ericson et al. (2006) was used as topological constraint.

The obtained phylogeny (Fig S1) was used un-manipulated together with the logarithmic values of U_e, body mass and wing loading from bird species selected to represent the endpoint of each branch in the phylogeny, to calculate independent contrast values according to Felsenstein (1985) and Garland et al. (1992) in the software PDAP (Maddison & Maddison 2004, Midford et al. 2005). Phylogenetic independent, reduced major axis regressions through origo were obtained using the standardized values of the independent contrasts (Fig S2). The 95% confidence intervals and Standard Error for the regression coefficients were calculated using 100,000 bootstrap iterations in the software SPSS 12.0.1.

References in this protocol:

Barker, F.K., Barrowclough, G.F. & Groth, J.G. (2002) A phylogenetic analysis for passerine birds: taxonomic and biogeographic implications of an analysis of nuclear DNA sequence data. *Proc. R. Soc. Lond. B* **269**: 295-308.

Barker, F.K., Cibois, A., Schikler, P., Feinstein, J. & Cracraft, J. (2004) Phylogeny and diversification of the largest avian radiation. *Proc. Natl Acad. Sci. USA* **101**: 11040-11045.

Ericson, P.G.P. (1997) Systematic relationships of the palaeogene family Presbyornithidae (Aves: Anseriformes). *Zool. J. Linnean Soc.* **121**(4): 429-483.

Ericson, P.G.P., Christidis, L., Cooper, A., Irestedt, M., Jackson, J., Johansson, U.S. & Norman, J.A. (2002) A Gondwanan origin of passerine birds supported by DNA sequences of the endemic New Zealand wrens. *Proc. R. Soc. B* **269**: 235-241.

Ericson, P.G.P., Anderson, C.L., Britton, T., Elzanowski, A., Johansson, U.S., Källersjö, M., Ohlson, J.I., Parsons, T.J., Zuccon, D. & Mayr, G. (2006) Diversification of Neoaves: integration of molecular sequence data and fossils. *Biol. Lett.* **2**: 543-547.

Felsenstein, J. (1985) Phylogenies and the comparative method. *Am. Nat.* **125**: 1-15.

Garland, T. Jr., Harvey, P.H. & Ives, A.R. (1992) Procedures for the analysis of quantitative data using independent contrasts. *Syst. Biol.* **41**: 18-32.

Lanave, C., Preparata, G., Saccone, C. & Serio, G. (1984) A new method for calculating evolutionary substitution rates. *J. Mol. Evol.* **20**: 86–93.

Livezey, B.C. (1997) A phylogenetic analysis of basal Anseriformes, the fossil Presbyornis, and the interordinal relationships of waterfowl. *Zool. J. Linnean Soc.* **121**(4): 361-428.

Maddison W.P. & Maddison D.R. (2004) Mesquite: A modular system for evolutionary analysis. Version 1.05. http://mesquiteprodject.org.

Midford P.E., Garland T. & Maddison W.P. (2005) PDAP Package of Mesquite.

Rodriguez, F., Oliver, J.L., Marin, A. & Medina, J. R. (1990) The general stochastic model of nucleotide substitution. *J. Theor. Biol.* **142**: 485–501.

Fig S1

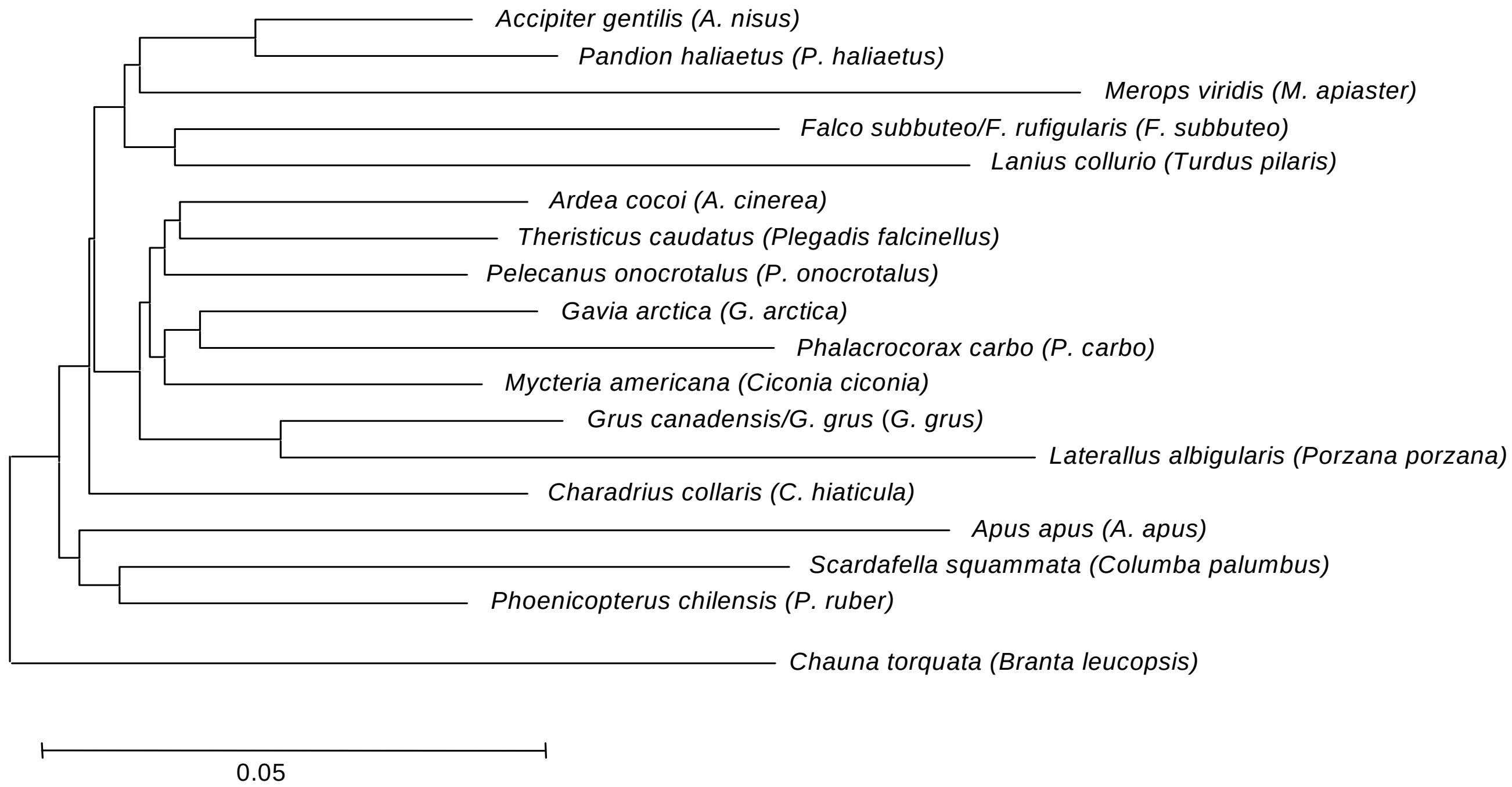

Figure S1 Phylogeny used in calculations of independent contrasts for U_e in relation to mass and wing loading. The tree includes 18 bird species from which DNA sequences of five nuclear genes have been obtained (Ericson et al. 2006), in two cases are genes from two close related species combined for the phylogenetic reconstruction. Taxon names follow Ericson et al. (2006), names of the corresponding taxon used in the contrasts analysis are displayed in brackets. Branch lengths were calculated using the maximum-likelihood criterion (–ln likelihood = 21692.4) and with the tree topology constrained to fit the higher-level systematic relationships in Neoaves recovered by Ericson et al. (2006). Bar represents 0.05 substitutions/site.

Fig S2

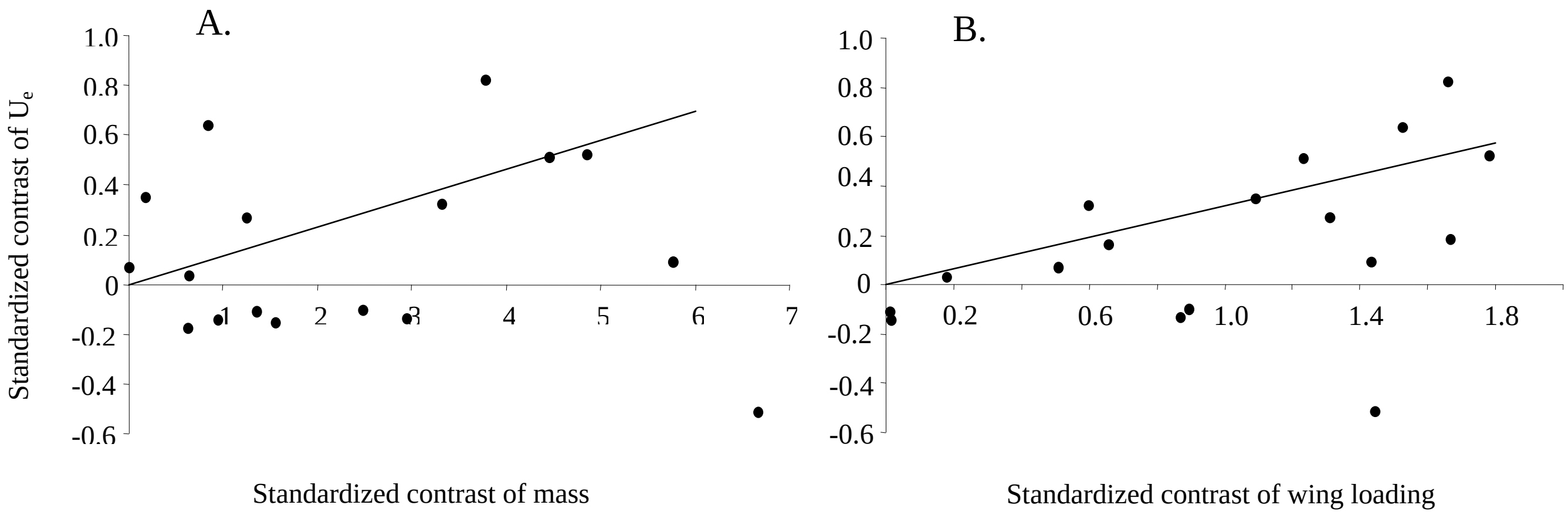

Figure S2 Phylogenetic independent, reduced major axis regressions through origo based on the standardized contrasts of the logarithmic values of U_e, body mass (A) and wing loading (B). Slope coefficients (A); 0.12, S.E.=0.024. (B); 0.32, S.E.= 0.041. The contrasts between 18 different representative bird species were calculated according to Felsenstein (1985) and Garland et al. (1992) in the software PDAP (Maddison & Maddison 2004, Midford et al. 2005). For phylogeny used to calculate the independent contrast se Fig S1.
